# Bisphenol-A: Epigenetic Reprogramming and Effects on Reproduction and Behavior

**DOI:** 10.3390/ijerph110707537

**Published:** 2014-07-22

**Authors:** Guergana Mileva, Stephanie L. Baker, Anne T.M. Konkle, Catherine Bielajew

**Affiliations:** 1 School of Psychology, University of Ottawa, 136 Jean Jacques Lussier, Vanier Hall, Ottawa, ON K1N 6N5, Canada; E-Mails: stephanie.baker@uOttawa.ca (S.L.B.); anne.konkle@uOttawa.ca (A.T.M.K.); catch@uOttawa.ca (C.B.); 2 Interdisciplinary School of Health Sciences, University of Ottawa, 25 University Private, Thompson Hall, Ottawa, ON K1N 6X1, Canada

**Keywords:** bisphenol A, endocrine disruptor, reproduction, epigenetics, toxicity, behavior

## Abstract

Bisphenol A (BPA) is a synthetic compound used in the production of many polycarbonate plastics and epoxy resins. It is one of the most widely produced chemicals in the world today and is found in most canned goods, plastics, and even household dust. Exposure to BPA is almost universal: most people have measurable amounts of BPA in both urine and serum. BPA is similar in structure to estradiol and can bind to multiple targets both inside and outside the nucleus, in effect acting as an endocrine disruptor. Research on BPA exposure has accelerated in the past decade with findings suggesting that perinatal exposure to BPA can negatively impact both male and female reproduction, create alterations in behavior, and act as a carcinogen. BPA can have both short term and long term effects with the latter typically occurring through epigenetic mechanisms such as DNA methylation. This review will draw on both human and animal studies in an attempt to synthesize the literature and examine the effects of BPA exposure on reproduction, behavior, and carcinogenesis with a focus on the potential epigenetic mechanisms by which it acts.

## 1. Introduction

Bisphenol-A (BPA) is one of the most ubiquitous synthetic chemical compounds in production today, with over three million tons produced annually [1]. BPA belongs to the bisphenol group of compounds that possess two hydroxyphenyl groups. Because of this chemical structure, BPA is extensively used in the production of polycarbonate plastics and epoxy resins. Common products made with BPA include water bottles, water piping, the lining of tin cans, toys, and thermal receipt paper (for review of BPA in polycarbonates see [2]). The extensive use of BPA means that environmental exposure is very common. Recent research indicates that in this everyday exposure BPA acts as an endocrine disrupting compound (EDC) even in low doses (for a review see [3]) [4].

BPA was originally synthesized in the late 19th century and subsequently promoted as a synthetic estrogen (xenoestrogen) after its estrogenic effects were discovered in the early 20th century [5]. At that time a structural analog of BPA, diethylstilbestrol (DES), replaced BPA in clinical use as it was found to be a much more potent synthetic estrogen [6]. DES was prescribed to millions of women around the world to prevent miscarriages. The daughters of these women went on to have a much higher likelihood of developing clear-cell adenocarcinoma of the vagina as well as general defects of the genital tract [7]. DES was consequently removed from the market and its production was completely halted in the late 1990s. Since it took nearly 20 years following prenatal exposure for the discovery of the malignant effects of DES, this raises the question of whether low level, chronic exposure to other EDCs, such as BPA, may also have deleterious effects which may only be detected on a longer time course and are harder to observe.

The goal of this article is to broadly cover the current literature regarding BPA modes and levels of exposure, as well as its actions at the cellular level. Further, the effects of BPA exposure during development with specific focus on behavior and reproduction will be addressed. Throughout this paper, the connection between BPA-induced epigenetic modifications will be discussed whenever possible.

## 2. Environmental Contamination

Direct release of BPA into the environment occurs during manufacturing and processing activities via release into air, water, soil, and through underground injection. According to the U.S. Environmental Protection Agency (EPA), the worldwide estimated release of BPA into the environment currently exceeds one million pounds per year [8]. Not surprisingly, BPA has been detected in indoor air, dust, soil, water (dinking, surface, and ground), sediment, municipal and industrial waste, and food (for a review see [9]). BPA also accounts for the majority of estrogenic activity detected in landfill leaching in North America [10] and Japan [11] based on tests on human cell lines. Under aerobic conditions, BPA appears to biodegrade quickly, as compared to other bisphenols, but under anaerobic conditions it does not degrade or does so very slowly [12]. However, recent studies suggest that certain strains (*i.e.*, *Bacillus*) are able to effectively biodegrade BPA at high rates (10 mg/dm^3^) under anaerobic conditions [13]. Therefore, even though promising research suggests that BPA can be biodegraded by certain types of bacteria, fungi, and algae [9], there is potential for environmental accumulation due to the substantial quantities released into the environment.

## 3. Human BPA Exposure

The primary source of BPA exposure in humans is thought to occur through ingestion; however, transdermal absorption [14] and inhalation of contaminated airborne dust [1] are probable secondary routes. BPA can readily leach into food sources through the epoxy resin linings of canned goods and those which have been in polycarbonate packaging [15,16]. While migration rates of BPA into water are higher at increased temperatures [15,17] significant migration can also occur at room temperature [18,19]. Because canned goods are both inexpensive and convenient, their use is very common in developed countries and a significant source of ingested BPA.

Reliable methods have been developed to quantify BPA concentrations in human urine, serum, and plasma (for a review see [20]). Large biomonitoring studies have found that more than 90% of U.S. [21] and Canadian participants [22] have detectable levels of BPA in their urine. A study of rural *vs.* urban girls in Egypt showed no difference in urinary BPA concentration which was attributed to the use of plastic containers to store food in both areas [23]. This also suggests that the gap between rural and urban living is closing and that BPA can be widely found in both areas. Carwile and colleagues reported that participants given canned soup for five consecutive days had their urine BPA levels increase more than 100% while no differences were found in participants who consumed fresh soup [24]. Conversely, urine levels of BPA significantly decreased after just three days of eating fresh foods that were not canned or packaged in plastic [25], indicating that a large number of prepared foods contain measurable amounts of BPA.

In the U.S., of 267 food products—such as beverages, dairy products, fats and oils, fish and seafood, cereals, fruits, vegetables and miscellaneous others—analyzed for BPA or its analogues, 75% were found to contain quantifiable levels of BPA, Bisphenol S (BPS) or Bisphenol F (BPF) [26]. In this study, condiments and other preserved foods had the highest level of bisphenols while fruits and beverages contained the least. In China, of 289 food samples from nine cities, 78% had bisphenols, with BPA being the most prevalent in over 60% of the samples [27]. In Canada, of 154 food samples, only 36% had quantifiable amounts of BPA, with the highest found in canned fish (101 ng/g) and corn (83.7 ng/g) [28]. The leaching of BPA and other chemicals are routinely underestimated or simply not stringently assessed [29]. The above research not only illustrates the ubiquitous nature of BPA as an environmental toxicant, it also explains why most of the general population has some measurable amount of BPA in their bodily fluids (*i.e.*, urine, serum, saliva *etc.*) [30].

According to the U.S. EPA, the current lowest-observable-adverse-effect level (LOAEL) of BPA is 50 mg/kg bodyweight per day. This level is based on a series of early studies measuring BPA induced weight change over generations in dogs, rats, and cats in the mid-1980s. To our knowledge, this level has remained unchanged since 1993 when it was first established, even in the face of mounting evidence that lower levels of BPA have physiological effects. In 2006, the European Food Safety Authority set their tolerable daily intake of BPA at 0.05 mg/kg body weight/day, significantly lower than that of the U.S. One reason that policy change in the U.S. is lagging could be because of early toxicokinetic studies of BPA and their findings. In addition, many of the biomonitoring studies mentioned above appear to be overlooked by the governing bodies that regulate BPA [30]. For this reason, a large part of the scientific community studying BPA and its adverse effects is in agreement that these toxicokinetic studies should not be the only ones used in policy making [31,32].

## 4. BPA Metabolism and Cellular Mechanism of Action

The toxicokinetics of BPA were first assessed in humans by Volkel and colleagues who administered a low dose (5 mg) of BPA orally to nine participants. They found that once ingested, BPA was quickly metabolized in the liver into bisphenol A-glucuronide, a highly soluble metabolite that is then eliminated through urine. It appeared that since bisphenol A-glucuronide has no hormonal activity, oral BPA metabolism deactivates the estrogenic activity of BPA and allows very little “free”, unconjugated, BPA to bind to estrogen receptors (ERs) [33]. This contention corroborated past studies in rats with orally administered BPA having the lowest bioavailability in comparison with intraperitoneal and subcutaneous injection of BPA [34]. In sum, early studies of human BPA metabolism suggested that BPA would have little to no effects in the body because of its rapid metabolism. However, there is mounting evidence that free BPA, even at low doses, can act as a potent EDC with lasting consequences.

Since BPA is a well-known xenoestrogen, its effects on estrogen receptors (ERs) have been extensively studied. ERs are important in the human body as they can carry out both genomic and non-genomic signal transduction (for a review see [35]). Genomic signal transduction is a slow process by which ERs regulate transcription and in turn gene expression. The non-genomic actions of ERs are very rapid and involve the modification of regulatory proteins. Until recently, endocrine disruption induced by BPA has been considered weak because compared to estradiol (E_2_), BPA concentrations need to be 1000 fold higher to induce the classic nuclear estrogen receptors ERα and ERβ [36]. Taken together, these studies propagated the view that exposure to BPA had little impact due to its low binding affinity to ERs and the fact that it is quickly inactivated and excreted.

In contrast, more recent basic research examining BPA exposure shows stark differences from the toxicokinetic studies. Animal studies examining perinatal BPA exposure have since reported that even doses significantly lower than those set by the U.S. EPA (50 mg/kg body weight/day) can cause significant changes in the brain and development (for review see [37]).

There are multiple reasons as to why previous human and animal studies have failed to report the low dose effects of BPA exposure. First, although the binding affinity of BPA for ERα and ERβ—the two most studied ERs—is very low, it is now clear BPA can and does bind multiple other targets within the nucleus and on the cell membrane and that the concentration of BPA needed to activate these targets is much lower than for ERα and ERβ (for a review see [38,39,40]. In fact, even at low doses, BPA can activate nuclear estrogen related receptor (ERR) γ and interact with the ligand domain of classic ERs [41]. BPA also binds to non-classical membrane estrogen receptors (mERs) creating rapid nongenomic changes that are insensitive to antiestrogens [42]. Furthermore, in pancreatic-α and -β cells, the binding sites of mERs show equal affinity for BPA, DES, and E_2_ with a concentration of 10^−9^ M BPA able to influence Ca^2+^ concentration oscillations [42,43]. In rats, BPA-induced alterations of ER expression in the hypothalamus occur in a sexually dimorphic manner [44]. This indicates that while the affinity of BPA for classic ER receptors is low, they readily bind to estrogen-related receptors. Binding of specific ERs by BPA metabolites can be at a rate 100 times higher than BPA itself [45]. BPA is also an antagonist to human androgen receptors (AR) as it binds to ARs, effectively inhibiting endogenous androgens [46,47] and can suppresses thyroid hormone receptor transcription in cell cultures thereby disrupting thyroid hormone signaling [39]. These findings all shed light on the widespread effects of BPA illustrating that unconjugated BPA can affect multiple signal transduction pathways.

A second reason why previous studies have not found low dose effects of BPA exposure may be because most of these focused primarily on the deactivated metabolite, bisphenol A-glucuronide. However, the amount of unconjugated, active BPA in human serum is around 4.4 µg/L indicating that not all BPA is metabolized [48]. BPA is transformed through oxidation into a variety of metabolites, many of which may have more significant physiological impact than the compound itself [49]. For instance, the BPA metabolite bisphenol A 3,4-quinone can form covalent bonds with and damage DNA, in turn contributing to hepatotoxicity [50]. The chemical mixtures, metabolites, and compounds produced when BPA in municipal drinking water mixes with chlorine have also been studied. At low concentrations (10^−15^–10^−7^ M), these chlorinated or sulfonated types of BPA can affect the binding affinity of ERα to other proteins [51]. BPS also acts as an EDC and can lead to alterations in cell death, proliferation, and mutagenicity [52,53]. This has important implications for human health, as it is commonly used as a replacement of BPA in plastic drinking bottles.

Furthermore, EDCs, like the natural hormones they frequently mimic, show non-monotonic dose response curves [54]. These non-monotonic relationships—defined as nonlinear dose-effect curves—can complicate the results obtained for effects of low and high dose administration. For instance, BPA exposure shows an inverted U-shaped dose response when female mice weight is assessed, while the opposite is true in male mice [55]. Specifically, low dose (1 µg/mL) exposure to BPA significantly increases female adipose tissue weight, while high dose (10 µg/mL) exposure has no effect. Conversely, weight was only affected at high doses of BPA exposure in male mice in the same study. This not only highlights the sexually dimorphic effects of BPA exposure, but also the non-linear dose response curves typical of EDCs. These non-monotonic relationships indicate that the effects of low and high dose exposure are not always constant between outcome measures and that studying only high dose responses, as is common in toxicological studies, is not sufficient.

Finally, many studies look only at global physical characteristics such as weight when assessing the effects of BPA exposure. More recently, a risk assessment study undertaken by the Biochemical Toxicology Division of the US Food and Drug Administration (FDA) has also reported that perinatal exposure to BPA causes adverse effects such as decreased pre- and post-natal weight only at very high doses (100,000 and 300,000 µg/kg body weight per day), significantly higher than the regulatory guidelines have set [56]. However, this may overlook more subtle changes in behavior or in the brain which were not measured.

Taken together there is now compelling evidence that BPA and its metabolites can not only bind to multiple target ERs, ERRs, mERs and ARs but that they can alter steroid synthesis and induce sexually dimorphic changes in the mammalian brain at doses significantly lower than that set by the EPA.

## 5. BPA and Epigenetic Modifications

In both clinical and preclinical research, BPA exposure has been linked to a variety of diseases. These include cardiovascular disease [57], diabetes [58], obesity [59,60], reproductive disorders [61], breast cancer [62], prostate cancer [63], hepatotoxicity [64], impaired synaptic remodeling (for review see [65]), and even depressive-like behavior in mice [66] and rats [67]. The mechanism by which BPA influences these diseases is not known, but evidence suggests that epigenetic processes play a role (for review see [68]).

Epigenetics is a large field of study regarding the molecular processes responsible for heritable and stable changes in gene expression that do not involve changes to the DNA sequence [69]. In essence, even though the DNA sequence remains fixed, the expression or silencing of genes and gene regions can be achieved via a variety of epigenetic mechanisms and in response to a variety of environmental exposures. DNA methylation is among the most studied mechanism of epigenetic regulation. Methylation is the process of adding a methyl group to the cytosine nucleotide within a cytosine-phosphate-guanine sequence of DNA, referred to as a CpG site. This can act to silence gene expression in that region of DNA [70].

In sexually reproducing organisms, when an embryo is fertilized it undergoes extensive germline reprogramming which “resets” or demethylates the DNA, creating a “clean slate” [71]. Whereas most DNA methylation is erased, some epigenetic marks can be retained across generations through a process called transgenerational epigenetic inheritance [72]. Though the mechanisms are as yet unclear, it appears that maternal or paternal phenotypes can be passed on via stable changes to offspring gene expression (for review see [73]) and that these stable epigenetic modifications can be influenced by environmental stimuli [74]. Furthermore, the mechanisms responsible for DNA methylation are known to respond to external stimuli during the perinatal period and could result in disease [75]. In other words, the external environment can trigger epigenetic change in parents that can then be passed on to the offspring in a non-random fashion. This epigenetic inheritance has been proposed as a potential mechanism in rapid adaptive evolution [74,76]. However, environmental contaminants such as EDCscan also affect both DNA methylation and epigenetic marks creating long lasting changes through subsequent generations (reviewed in [77,78]).

Environmental contaminants such as mercury, lead, arsenic, and BPA have all been shown to alter DNA methylation in humans [79,80]. Hypermethylation was found in tail tissue of mouse offspring perinatally exposed to very low doses of BPA (5 ng/kg) [81]. In neonatal rats, BPA exposure has been linked to altered DNA methylation at key cell signaling genes, increasing the likelihood of developing precancerous prostatic lesions in adulthood [82,83]. Neonatal BPA exposure causes alterations to the epigenome in the frontal brain of mice [84]. These studies highlight the importance of analyzing the effects of BPA exposure in human and non-human mammalian health. The mechanisms through which BPA exposure influences pathology also need to be examined in depth.

## 6. BPA Exposure During Development

The growing organism is most susceptible to the negative consequences associated with endocrine disruption. Fetuses, infants, and young children thus represent a high risk group that is exposed to environmental contaminants at higher levels than adults due to differences in diet (formula feeding from bottles), behavior (such as mouthing plastic toys), and metabolic and physiological characteristics [85]. The perinatal period is one of the most important periods of neurodevelopment in which the external environment plays a significant role in programming and re-programming gene expression.

In pregnant rats, the liver’s ability to metabolize free BPA to its deactivated state (BPA-glucuronide) is attenuated to 60% compared with that of non-pregnant female rats [86]. These animal studies indicate that BPA is not excreted as readily during pregnancy and might create a chronic level of BPA exposure. One human study supports this hypothesis: urine BPA concentration increased by 26% of baseline (or compared with pre-pregnancy levels) in women when they became pregnant [87].

Studies of pregnant women, and in some cases their fetuses, have indicated that exposure to BPA is nearly ubiquitous, especially in developed countries. A fairly small Australian study (N = 26) showed measurable amounts of BPA in 85% of their sample of pregnant women with a median of 2.41 µg/L [88]. In a Canadian study of pregnant women, BPA levels ranged from non-detectable to almost 5 ng/mL, with this value highly correlated to that of their fetus [89]. Another large study of pregnant women in Canada between 2008–2011 found that 88% had measurable BPA levels in their urine and that increasing maternal age, not smoking, and higher education and income correlated with a decrease in BPA levels [90]. These demographic correlations with BPA levels seem to be consistent with findings from other countries such as Spain [91] and the U.S. [92].

Free BPA has also been detected in early and late pregnancy serum [93], maternal plasma at birth, and placental tissue [94]. A placental perfusion experiment using human full term placenta, indicated rapid transfer of an environmentally relevant, low dose (10 ng/mL) of BPA from the maternal placental unit to the fetal unit [95]. BPA in the fetal compartment began to rise within 10 min and reached 27% of the maternal dose within 180 min, the monitoring endpoint [95]. A similar study supports rapid BPA transfer in human perfused placenta, placental explants and in skin diffusion experiments [96]. It is now clear that most pregnant women have measurable amounts of BPA in their bodies, a concern not only to them, but also to their growing fetuses.

## 7. Perinatal BPA Exposure—Reproduction

Environmental contaminants can have long lasting effects on fertility. It is well established that exposure to low doses of BPA can trigger changes in both male and female reproductive physiology. In one of the first studies of its kind, vom Saal and colleagues found that the male offspring of pregnant mice who had been fed BPA at 20 ng/g bodyweight (20 parts per billion) had decreased sperm production, while those fed 2 ng/g bodyweight (2 parts per billion) had a permanent increase in the pheromone-producing preputial glands. The effects of BPA exposure on reproductive organs can have evolutionary consequences by decreasing individual fitness, and affecting population dynamics [97]. Globally, there is worry that human fertility rates and fecundity are declining, especially in developed countries [98], but measuring these rates is very difficult and imprecise [99]. While many factors might influence these declining rates—including increased rates of obesity, decreased sperm count, and delayed pregnancy—the effect of xenoestrogens such as BPA cannot be ignored. Both animal work and human correlational data looking at urine BPA concentration in association with both male and female fertility measures are outlined below.

### 7.1. Non-Human Mammalian Fertility

In male animals, exposure to environmental contaminants can impact the reproductive organs and in turn fertility (for a review see [100]). For instance, the common EDCs vinclozolin (an anti-androgenic fungicide) and methoxychlor (an estrogenic pesticide) have been examined for their activity on male fertility. In rats, transient exposure to methoxyclor (200 mg/kg/day) during gestation was reported to increase male infertility via epigenetic reprogramming in all subsequent generations (F1 to F4) [101]. Gestational exposure to vinclozolin (100 mg/kg/day) was found to cause long lasting effects in reproductive organs (prostate, testes) as well as immune and kidney function in males from F1 to F4 [102]. These were some of the first studies to find a transgenerational effect of EDCs on fertility and disease. In a series of studies by Manikkam, epigenetic changes in the F3 offspring were found after gestational exposure of the F0 dam to a pesticide mixture [103], dioxin [104], and a plastic mixture containing BPA [105] created significant sperm epimutations in F3 animals.

Research in rats has shown that BPA exposure and its effects on reproductive hormones can cause permanent changes in the entire male hypothalamic-pituitary-gonadal axis [106]. Perinatal exposure to BPA can create transgenerational changes in the number of testicular steroid hormone receptors, sperm motility, and sperm count (for a review see [107]). The fact that F1, F2, and F3 offspring all show these alterations is indicative of permanent changes to the germline. These epigenetic changes could be due to DNA methylation abnormalities in the testis of the adult offspring [108]. However, it is not only perinatal BPA exposure that can affect the male rat germ cells. A recent study by Tiwari and Vanage (2013) showed that exposing adult male rats to 5 mg/kg bodyweight BPA over a period of six days will create dominant lethal mutations in germ cells. These lead to reduced sperm production and motility [109]. It is therefore important to examine the epigenetic changes induced by BPA exposure not only in early life but also in adulthood as both appear to be influential in male fertility.

Environmental contaminants do not only affect male fertility. In female animals, exposure to a common compound found in the insect repellent called *N*,*N*-Diethyl-3-methylbenzamide (DEET) and dioxins promotes transgenerational early-onset puberty [110]. *In utero* exposure to low doses of BPA (20 µg/kg) in mice causes vaginal opening to occur significantly earlier than in control animals [111] whereas exposure during adulthood significantly alters both uterine weight and pregnancy rates (54% treated *vs.* 100% controls) [112]. Both fertility and fecundity were significantly decreased when pregnant mice were administered 25 µg/kg of BPA subcutaneously through osmotic pumps [113]. Finally, neonatal exposure to high doses of BPA (500 µg/50 mL) has been reported to alter ovarian morphology, increasing the number of cysts and creating a polycystic ovarian syndrome phenotype in female animals [114]. The mechanism by which BPA can affect female reproductive capacity is still unclear, but research suggests that BPA can disrupt early oocyte formation through its actions on ERβ [115].

In primates, Calhoun *et al*. showed that exposing pregnant rhesus macaques to BPA (400 µg/kg/day) during the third trimester, but not before, can alter transcription and expression of developmental genes. These changes in gene expression are likely to affect uterine function later in life [116]. BPA exposure late in gestation can not only decrease genome-wide DNA methylation in the placenta (at 50 µg/kg exposure) but also disrupts imprinted gene expression [117]. These alterations in DNA methylation, and in turn gene expression, are indicators of BPA-induced epigenetic reprogramming that can lead to infertility, decreased fecundity, and affect development as a whole. Notably, maternal supplementation with folic acid in mice appears to reverse BPA-induced epigenetic modification in offspring [118]. Taken together, these animal studies provide extensive evidence of the negative effects of low-dose perinatal or adult BPA exposure on fertility.

### 7.2. Human Fertility

Exposure to EDCs can also negatively impact men’s reproductive health (for review see [119]). To our knowledge no studies exist showing a causal relationship between BPA exposure and decreased fertility in humans. Much research has focused on the correlation between urine and serum BPA and hormone concentrations. In a Danish study by Lassen *et al*., 98% of men (median age 19 years) tested had measurable amounts of BPA in their urine with a median concentration of 3.25 ng/mL [120]. Men in the highest quartile for urine BPA had significantly higher levels of E_2_, testosterone, and luteinizing hormone as compared to those in the lowest quartile. Moreover, these men also had a significant decrease in sperm motility [120]. Urinary BPA concentration showed a modest, though not significant, relationship with a reduction in sperm count and altered morphology [121].

In women, serum concentrations of BPA and Bisphenol B are associated with an increased likelihood of endometriosis [122], although these findings are not consistent [123]. Other studies link blood BPA levels with polycystic ovarian syndrome [124] and suggest that the positive correlation between androgen concentration and serum BPA could be a cause [125]. Increasing urine BPA concentration has also been associated with a higher implantation failure, decreased ovarian response, a decrease in blastocyst formation, and recurrent miscarriage [126,127,128]. Preliminary evidence in a small sample of pregnant women in Mexico also suggests that there may be a positive correlation between urine BPA concentration and premature birth [129].

Multiple associations have been found between BPA concentration in serum and urine in women undergoing *in vitro* fertilization (IVF). These findings corroborate other measures assessing BPA’s effect on fertility. For example, a significant relationship between the number of oocytes retrieved, peak E_2_ levels, and increasing BPA in urine has been found in women seeking fertility treatment [130]. In a similar study, while no relationship was found between the number of oocytes retrieved and serum BPA concentration, there was an association between serum BPA concentration and a reduced E_2_ response during IVF [131]. E_2_ is typically an indicator of follicular response during IVF and these BPA-induced changes in E_2_ may render gonadotropin fertility treatments ineffective. While the above is based on correlational data, these links can give fertility treatment clinics another biomarker to examine and potentially control for to make IVF more effective. The nature of human studies is such that drawing conclusions about the effect of BPA exposure on fertility is often not possible, given the wide range of contradictory results. However, a combined approach using animal, toxicokinetic, endocrinology, and epidemiological studies can help to better understand the true effects of BPA exposure.

## 8. Perinatal BPA Exposure—Behavior

### 8.1. Non-Human Mammalian Behavior

Since BPA can bind to multiple sex hormone receptors it has the potential to modify endogenous hormone concentrations. Even slight changes in the concentrations of hormones can create alterations in the development of sexually dimorphic brain nuclei and the behaviors they underlie. Some of the well-characterized sexually dimorphic nuclei are located in the hypothalamus and are differentially affected by circulating steroid hormones such as E_2_ and testosterone which also affect behavioral outcomes (for a review see [132]). In particular, early life treatment with exogenous E_2_ or testosterone increases apoptosis in the anteroventral periventricular nucleus, a structure many times larger in females, resulting in a masculinized female brain [133,134]. Since BPA passes freely from mother to child through the placenta and breast milk, and because the perinatal period is crucial for brain development, BPA exposure during this time might disrupt the normal process of masculinization/feminization of the brain and in turn disrupt behavior. In fact, rats perinatally exposed to BPA (1.5 mg/kg/day) show altered sexual differentiation of the locus coeruleus, a nucleus directly involved in the stress response [135]. In rats, BPA-induced alterations of ER expression also occur in the hypothalamus in a sexually dimorphic manner [44].

Maternal exposure to EDCs has been linked to changes in sexual behavior in both male and female rats. Early studies of the effects of the estrogenic pesticides DDT and methoxychlor and DES on behavioral outcomes in male mice found that prenatal exposure to low doses of all three compounds increased their territorial behavior [136]. Exposing rats to 40 µg/kg/day of BPA during pregnancy and lactation also increased defensive behavior in male offspring while increasing sexual motivation and receptivity in female offspring [137]. *In utero* BPA exposure in mice eliminated sex-typical novelty responses and exploration in the open-field test in adulthood [138] and masculinized play behavior in female rats [139]. Low dose gestational exposure (20 µg/day) to BPA can also create transgenerational epigenetic changes in social behavior by decreasing the mRNA for vasopressin and oxytocin, two peptides important in the expression of social behavior [140].

BPA exposure has also been linked to sexually dimorphic alterations in anxiety-like behavior and general motor activity [141]. In rat behavioral research, female animals typically show less anxiety and depressive-like behaviors in measures such as the elevated plus maze and forced swim tests, respectively [142,143]. Research by Jones and Watson found that perinatal exposure of Long-Evans rats to BPA at 5 µg/kg bodyweight, but not above, eliminates sex differences in the elevated plus maze and forced swim test between males and females. This is indicative of BPA’s actions on emotional behavior in a dose dependent and sexually dimorphic manner proposed to be through a demasculization of typical male behavior [144]. Notably, BPA exposure did not affect learning and memory in the Morris water maze [144]. However, an increase in depressive-like behavior in the forced swim after low dose prenatal BPA exposure (~15 µg/kg/day) was reported in male, but not female, Wistar rats [67]. These alterations in anxiety and depressive-like behaviors could be mediated by circulating corticosterone or changes in glucocorticoid receptors (GRs) in key brain structures such as the hippocampus. For instance, in utero exposure to low dose BPA (40 µg/kg body weight) in male and female Wistar rats significantly increases only female serum corticosterone levels while reducing GR levels in the hippocampus [145]. However, following a mild stressor there were sexually dimorphic changes in both serum corticosterone and GR receptors as well as general spatial recognition impairments in both male and female BPA exposed animals [145].

The findings in behavioral studies can be influenced by the use of a wide variety of strains, methods, and species. There are known sex and strain differences in depressive-like behavior in rats exposed to depressogenic stimuli associated with the chronic mild stress procedure, for example. This indicates that both the response to and outcome of stressful stimuli is sexually dimorphic and dependent on strain [146,147]. Therefore, it is important to determine if there are sexually dimorphic changes in brain and behavior after BPA exposure in multiple strains of animals. Taken together, the above studies show that BPA-mediated changes in the brain can create long lasting behavioral alterations even at doses significantly lower than those delineated by the EPA (50 mg/kg bodyweight).

### 8.2. Human Behavior

It is often difficult to study the effects of contaminants through the human lifespan, especially with regards to behavior. This is due to the large number of possible confounding factors and the relative variability in human subjects. For this reason, there are few studies on the effects of BPA exposure on human behavior. However, a small number of longitudinal studies have been able to provide evidence for the potential negative effects of early life BPA exposure while cross-sectional approaches have evaluated urine BPA concentration with direct behavioral observation. These studies are correlational in nature and often present with multiple limitations but are a necessary next step in examining the effects of BPA on human behavior.

In a recent longitudinal study, maternal BPA urine concentration during pregnancy was linked with increased anxiety and depression in boys, but not girls, at age seven [148] and is consistent with previous studies [149]. However, studies from different groups show that prenatal BPA exposure affects girls, but not boys, in emotionality and relational domains [150,151]. In cross-sectional analyses, BPA concentrations in childhood spot urine are associated with increased internalizing and attention problems in boys and externalizing and conduct problems in girls at age seven [148]. Furthermore, a study observing children ages 8–11 found a positive association between BPA levels in urine and anxiety and depression as measured using the Child Behavior Checklist [152]. In the same study, a negative association was found between urinary BPA concentration and total learning quotient using the Learning Disability Evaluation Scale; however, no gender differences were observed. While the variation in the findings across studies is puzzling, it is clear that perinatal and childhood BPA exposure may alter behavior, perhaps in a sexually dimorphic manner, which agrees with the results of animal studies.

## 9. BPA-Induced Carcinogenesis

Exposure to EDCs during the perinatal period can create deleterious epigenetic modifications and, in some cases, trigger carcinogenesis [153]. It is now clear that xenoestrogens can and do negatively affect the fetus, exemplified by the multigenerational carcinogenic effects of DES. Mounting evidence suggests that BPA should also be classified as a carcinogen within the definitions established by the EPA. Notably, perinatal BPA exposure has recently been found to act as a carcinogen in the liver, increasing the incidence of hepatic tumors in 10 month old mice [154]. While the mechanism remains largely unknown, new research suggests that BPA exposure can influence motility in rat liver epithelial cells perhaps thought the induction of lysophosphatidic acid (LPA) G protein-coupled receptors [155]. The multiple isoforms of the LPA receptor are important in many cellular functions including cell proliferation and migration (For review see [156]) and are also implicated in tumorigenesis and resistance to anti-cancer drugs in certain cancer cell lines [157,158]). Taken together, it appears that BPA can affect LPA receptors important in key cellular functions and in turn may be one of the underlying mechanisms in BPA-induced tumorigenesis.

However, because of its effect as a xenoestrogen, the carcinogenic effects of BPA have been studied most often with respect to reproductive organs such as the mammary glands, ovaries and testes. Perinatal exposure to BPA at environmentally relevant levels in rats has been linked to mammary carcinogenesis in the rat dams (for review see [159]) and mammary gland adenocarcinoma in the female offspring as early as post natal day 90 [160]. Surprisingly, morphological changes in the mammary gland were also found in male mice offspring, implying that fetal BPA exposure could be a cause of gynecomastia, or the enlargement of mammary tissue seen in males [161].

Extrapolating to a human model, the above studies suggest that BPA’s effects as a xenoestrogen can alter the morphogenesis of the fetal mammary gland with the potential to create breast cancer in females and gynecomastia in males through epigenetic gene expression modification. The mechanism by which BPA might influence breast cancer in humans is so far unknown; however, Fernandez *et al*. (2012) showed that in human breast cancer cell lines, BPA increases DNA repair gene expression including BRCA1 and BRCA2. They concluded that women who have mutations in these two genes may be particularly susceptible to the negative effects of environmental BPA exposure [162].

BPA exposure has also been linked to the development of prostate carcinogenesis in both rats [163] and human prostate epithelium [63]. In 2014, an epigenetic mechanism for BPA-induced prostate cancer was proposed that involves increased prostate stem-progenitor cell self-renewal as well as upregulated expression of stem-cell related genes [63]. In other words, human prostate stem-progenitor cells appear to be directly affected by BPA. While other mechanisms may exist, there is no doubt that the epigenetic effects of BPA on the binding, synthesis, and metabolism of natural estrogens plays a large role. These studies illustrate the potent effects of EDCs, with special focus on BPA during the perinatal period and provide solid evidence that BPA can be classified as a carcinogen based on its role in both breast and prostate carcinogenesis.

## 10. BPA Regulatory Debate

Many endocrinologists believe that governments should use the precautionary principle to create stringent regulation in the face of multiple studies showing the low dose effects of EDCs in both animal and human research [4]. The precautionary principle itself is controversial, as it states that harmful substances should be strictly regulated even if there are gaps in the scientific knowledge about them. Up to this point, many countries including France, Sweden, and Turkey have banned the use of BPA in children’s food containers but, to our knowledge, only Canada has both banned BPA from baby bottles and declared BPA a toxic substance. According to Vandenberg (2011), by declaring BPA a toxic substance, Health Canada has invoked the precautionary principle, but more stringent legislation is yet to follow [164].

Even in the face of mounting evidence, the effects of low dose BPA exposure still remain controversial impeding regulatory policy. The debate on BPA safety, and its eventual resolution, will have wide ranging consequences not only on public health and private industry but also on the global economy. This is partially due to the ubiquitous nature of BPA in worldwide plastic and resin production and the public safety information supplied by plastics companies [165]. Michaels reported that when the American Plastics Council hired the Harvard Center for Risk Analysis to review animal studies evaluating the low-dose effects of BPA, they inspected 19 papers and found no consistent effects. This was highly contrasted by government funded research in which 94 out of 104 studies found significant low-dose effects [166]. Michaels further illustrated the concept of “manufactured uncertainty” using the BPA safety debate as an example. Manufactured uncertainty is defined as the ability of corporations to create public doubt in the scientific evidence behind regulatory policy [166]. Because BPA grosses approximately $6 billion USD a year, plastics companies often hire their own experts to inform policy makers by discrediting independent research [167]. For instance, 60 of the 80 studies which were to be used in regulatory policy were considered flawed by a plastics company expert [167]. In addition, weight-of-evidence studies conducted in 2006 and updated in 2009 reviewed a large number of studies and found no low-dose effects of BPA; there may be conflict of interest issues that underlie these interpretations [168,169,170].

Independent research in the late 1990s attempted to replicate findings from the vom Saal laboratory which showed significant low dose effects of BPA exposure [171,172]. However, these studies found no effect of low dose BPA and DES on prostate size and sexual maturation in both rats and mice [173,174,175]. The methodology employed by these early studies to look at low-dose effects of BPA has been highly scrutinized, potentially invalidating studies which found effects and those which did not [176]. Since then, the methodology used to measure BPA has been standardized by multiple researchers from different institutes. Furthermore, the National Institute of Environmental Health Studies, in association with the National Toxicology Program has set up funding for researchers working on low dose effects of BPA which so far include 223 studies between 2010 and February 2014 [177]. This paints a positive picture of collaboration between government researchers and regulatory agencies for the future of BPA research.

## 11. Conclusions

In this article the adverse effects of BPA are highlighted with a special focus on reproduction, behavior, and carcinogenesis and the potential epigenetic mechanisms by which BPA acts. There is strong evidence that the estrogenic and anti-androgenic effects of BPA can negatively impact brain and reproductive organ development by binding to multiple receptor targets both in and out of the nucleus. From altering the normal trajectory of sexual dimorphic brain development to reducing sperm count and motility, it is clear that low-dose BPA exposure, especially during the perinatal period, can have significant negative outcomes in later life. While correlational research shows links between BPA exposure and human behavior and disease, more research is needed to determine if the mechanisms by which BPA induces changes in non-human animals can be extrapolated to humans.
